# Hydroxonium triaqua­bis(biuret-κ^2^ *O*,*O*′)dichloridolanthanum(III) dichloride dihydrate

**DOI:** 10.1107/S1600536808010349

**Published:** 2008-04-18

**Authors:** William T. A. Harrison

**Affiliations:** a Department of Chemistry, University of Aberdeen, Meston Walk, Aberdeen AB24 3UE, Scotland

## Abstract

In the title compound, (H_3_O)[LaCl_2_(C_2_H_5_N_3_O_2_)_2_(H_2_O)_3_]Cl_2_·2H_2_O, the La atom is bonded to seven O atoms (arising from two *O*,*O*′-bidentate biuret mol­ecules and three water mol­ecules) and two chloride ions in an irregular arrangement. A network of N—H⋯O, N—H⋯Cl, O—H⋯O and O—H⋯Cl hydrogen bonds helps to establish the packing, leading to a three-dimensional network. The La atom, one Cl atom and four O atoms lie on a crystallographic mirror plane.

## Related literature

For related structures, see: Carugo *et al.* (1992 ▶); Rogers *et al.* (1993 ▶); Su *et al.* (2006 ▶); Haddad (1987 ▶, 1988 ▶); Harrison (2008*a* ▶,*b* ▶). For related literature, see: Brese & O’Keeffe (1991 ▶).
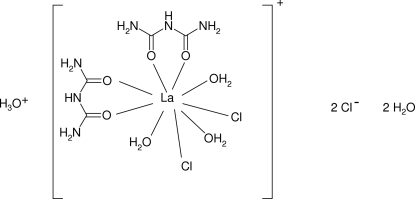


## Experimental

### 

#### Crystal data

(H_3_O)[LaCl_2_(C_2_H_5_N_3_O_2_)_2_(H_2_O)_3_]Cl_2_·2H_2_O
*M* *_r_* = 596.00
Orthorhombic, 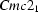

*a* = 17.6252 (7) Å
*b* = 6.8868 (3) Å
*c* = 17.0447 (7) Å
*V* = 2068.91 (15) Å^3^
*Z* = 4
Mo *K*α radiation
μ = 2.63 mm^−1^
*T* = 293 (2) K
0.30 × 0.23 × 0.17 mm

#### Data collection

Bruker SMART1000 CCD diffractometer
Absorption correction: multi-scan (*SADABS*; Bruker, 1999 ▶) *T*_min_ = 0.486, *T*_max_ = 0.636
11912 measured reflections
3793 independent reflections
3716 reflections with *I* > 2σ(*I*)
*R*_int_ = 0.018

#### Refinement

*R*[*F* ^2^ > 2σ(*F* ^2^)] = 0.016
*wR*(*F* ^2^) = 0.042
*S* = 1.09
3793 reflections
121 parameters
1 restraint
H-atom parameters constrained
Δρ_max_ = 0.70 e Å^−3^
Δρ_min_ = −0.67 e Å^−3^
Absolute structure: Flack (1983 ▶), 1805 Friedel pairs
Flack parameter: 0.001 (9)

### 

Data collection: *SMART* (Bruker, 1999 ▶); cell refinement: *SAINT* (Bruker, 1999 ▶); data reduction: *SAINT*; program(s) used to solve structure: *SHELXS97* (Sheldrick, 2008 ▶); program(s) used to refine structure: *SHELXL97* (Sheldrick, 2008 ▶); molecular graphics: *ORTEP-3* (Farrugia, 1997 ▶); software used to prepare material for publication: *SHELXL97*.

## Supplementary Material

Crystal structure: contains datablocks I, global. DOI: 10.1107/S1600536808010349/fj2113sup1.cif

Structure factors: contains datablocks I. DOI: 10.1107/S1600536808010349/fj2113Isup2.hkl

Additional supplementary materials:  crystallographic information; 3D view; checkCIF report

## Figures and Tables

**Table 1:** Selected bond lengths (Å)

La1—O3	2.503 (2)
La1—O2	2.5313 (13)
La1—O1	2.5318 (14)
La1—O4	2.542 (2)
La1—O5	2.562 (2)
La1—Cl1	2.9606 (4)

**Table 2:** Hydrogen-bond geometry (Å, °)

*D*—H⋯*A*	*D*—H	H⋯*A*	*D*⋯*A*	*D*—H⋯*A*
N1—H1⋯Cl2^i^	0.86	2.94	3.643 (3)	141
N1—H2⋯Cl1^ii^	0.86	2.83	3.574 (3)	145
N2—H3⋯Cl1^ii^	0.86	2.28	3.1399 (14)	173
N3—H4⋯O7^ii^	0.86	2.12	2.927 (3)	156
N3—H5⋯Cl2	0.86	2.75	3.3835 (18)	132
O3—H6⋯Cl2^i^	0.85	2.28	3.1181 (16)	168
O4—H7⋯Cl2^iii^	0.84	2.32	3.1396 (17)	164
O5—H8⋯Cl1^iv^	0.75	2.44	3.1566 (17)	160
O6—H9⋯O2^iii^	0.88	2.23	3.044 (3)	153
O6—H10⋯O7	0.86	2.35	2.941 (3)	126
O6—H10⋯O7^v^	0.86	2.35	2.941 (3)	126
O7—H11⋯Cl2^vi^	0.86	2.48	3.264 (3)	151
O7—H12⋯O1^vii^	0.84	2.09	2.922 (2)	168

Symmetry codes: (i) 
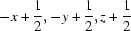
; (ii) 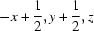; (iii) 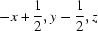; (iv) 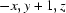; (v) 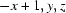; (vi) 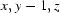; (vii) 
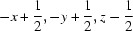
.

**Fig. 1.**
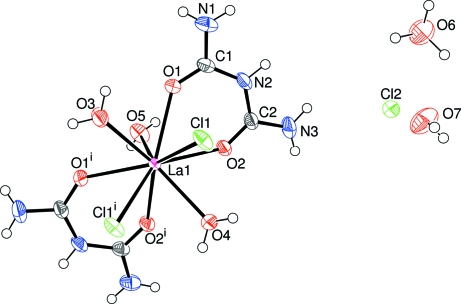
View of the molecular structure of (I) showing 50% displacement ellipsoids (arbitrary spheres for the H atoms). Symmetry code: (i) -x, y, z.

**Fig. 2.**
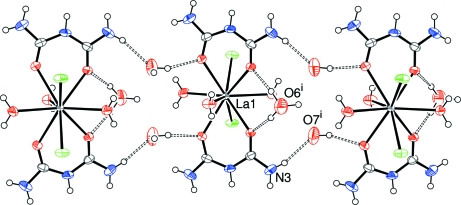
Fragment of the packing for (I) displaying the hydrogen bonds (shows an double dashed lines) leading to chains arising from the complex cation, the hydroxonium ion and the uncoordinated water molecule. Symmetry code: (i) 1/2 - x, 1/2 + y, z.

## supplementary crystallographic information

### Comment

No complexes of lanthanum(III) with biuret (biur), H_2_N—CO—NH—CO—NH_2_ (or C_2_H_5_N_3_O_2_) have been structurally characterized. The structures of two samarium-biuret complexes, Sm(biur)_4_.(NO_3_)_3_ (Haddad, 1987) and Sm(biur)_4_.(ClO_4_)_3_ (Haddad, 1988) have been described. In both cases, an SmO_8_ square antiprismatic coordination arises for the metal ion. Based on X-ray photographs, it was suggested that all the Ln(biur)_4_.(NO_3_)_3_ and Ln(biur)_4_.(ClO_4_)_3_ compounds are isostructural with their samarium prototypes. In this paper, we describe the synthesis and structure of the title compound, (I), in which three different ligands are bonded to the trivalent cation.

Compound (I) is an ionic salt containing a new [La(biur)_2_(H_2_O)_5_Cl_2_]^+^ complex ion. The complete cation is generated by crystallographic mirror symmetry, with La and the three water O atoms lying on the reflecting plane. A hydroxonium cation (with its O6 atom with site symmetry m), an uncoordinated chloride ion (Cl2) and an uncoordinated water molecule (O7) complete the structure (Fig. 1) of (I).

The resulting LaO_7_Cl_2_ polyhedral geometry in (I) (Table 1) can only be described as irregular. The Brese & O'Keeffe (1991) bond-valence sum for La1 in (I) of 3.29 is significantly larger than the expected value of 3.00. A local LaO_7_Cl_2_ grouping has been seen in various other compounds, including [LaCl_2_(H_2_O)(C_12_H_24_O_6_)]^+^.Cl^-^ (Rogers *et al.*, 1993) and [La(H_2_O)_4_Cl(C_3_H_7_O_3_]^2+^.2Cl^-^.H_2_O (Su *et al.*, 2006), but otherwise these phases have no similarity to (I).

The O,*O*-bidenate coordination of the biuret molecule to the lanthanum ion in (I) results in a six-membered chelate ring that is non-planar. As noted previously (Carugo *et al.*, 1992), the biuret molecule can be regarded as two planar amide fragments linked by the NH bridge. Here, the dihedral angle betwen the N1/C1/O1/N2 and N2/C2/O2/N3 units is 5.06 (10)°. The lanthanum cation deviates from the N1/C1/O1/N2 and N2/C2/O2/N3 mean planes by 0.894 (4)Å and 0.606 (4) Å, respectively.

The component species in (I) are linked by a dense array of N—H···O, N—H···Cl, O—H···Cl and O—H···O hydrogen bonds (Table 2) resulting in a three-dimensional network. Of note are the [001] chains resulting from the O—H···O hydrogen bonds involving the complex cation, H_3_O6 and H_2_O7 (Fig. 2).

The structure of (I) is different to those of the recently reported (Harrison, 2008*a*,b) *M*(biur)_2_(H_2_)_4_.Cl_3_ (*M* = Gd, Y) phases, perhaps because the larger La^3+^ cation can accommodate nine atoms in its coordination sphere.

### Experimental

0.1 *M* Aqueous solutions of LaCl_3_ (10 ml) and biuret (10 ml) were mixed and a small quantity of dilute hydrochloric acid was added, to result in a colourless solution. Colourless blocks of (I) grew over several days as the water slowly evaporated.

### Refinement

The N-bound hydrogen atoms were geometrically placed (N—H = 0.88 Å) and refined as riding with *U*_iso_(H) = 1.2*U*_eq_(N). The water and hydroxonium H atoms were located in difference maps and refined as riding in their as-found relative positions with *U*_iso_(H) = 1.2*U*_eq_(O). Although a plausible hydrogen bonding scheme results, some of the peaks were barely above the noise level of the data, and thus the positions of the O-bonded H atoms should be regarded as less certain.

### Crystal data

**Table:**

(H_3_O)[LaCl_2_(C_2_H_5_N_3_O_2_)_2_(H_2_O)_3_]Cl_2_·2H_2_O	*F*_000_ = 1176
*M**_r_* = 596.00	*D*_x_ = 1.913 Mg m^−^^3^
Orthorhombic, *C**m**c*2_1_	Mo *K*α radiation λ = 0.71073 Å
Hall symbol: C 2c -2	Cell parameters from 4771 reflections
*a* = 17.6252 (7) Å	θ = 2.3–32.5º
*b* = 6.8868 (3) Å	µ = 2.63 mm^−^^1^
*c* = 17.0447 (7) Å	*T* = 293 (2) K
*V* = 2068.91 (15) Å^3^	Block, colourless
*Z* = 4	0.30 × 0.23 × 0.17 mm

### Data collection

**Table:**

Bruker SMART1000 CCD diffractometer	3793 independent reflections
Radiation source: fine-focus sealed tube	3716 reflections with *I* > 2σ(*I*)
Monochromator: graphite	*R*_int_ = 0.018
*T* = 293(2) K	θ_max_ = 32.5º
ω scans	θ_min_ = 2.3º
Absorption correction: multi-scan(SADABS; Bruker, 1999)	*h* = −26→26
*T*_min_ = 0.486, *T*_max_ = 0.636	*k* = −10→9
11912 measured reflections	*l* = −24→25

### Refinement

**Table:**

Refinement on *F*^2^	Hydrogen site location: inferred from neighbouring sites
Least-squares matrix: full	H-atom parameters constrained
*R*[*F*^2^ > 2σ(*F*^2^)] = 0.016	*w* = 1/[σ^2^(*F*_o_^2^) + (0.0242*P*)^2^] where *P* = (*F*_o_^2^ + 2*F*_c_^2^)/3
*wR*(*F*^2^) = 0.042	(Δ/σ)_max_ < 0.001
*S* = 1.09	Δρ_max_ = 0.70 e Å^−^^3^
3793 reflections	Δρ_min_ = −0.67 e Å^−^^3^
121 parameters	Extinction correction: none
1 restraint	Absolute structure: Flack (1983), 1805 Friedel pairs
Primary atom site location: structure-invariant direct methods	Flack parameter: 0.001 (9)
Secondary atom site location: difference Fourier map	

### Special details

**Table:**

Geometry. All e.s.d.'s (except the e.s.d. in the dihedral angle between two l.s. planes) are estimated using the full covariance matrix. The cell e.s.d.'s are taken into account individually in the estimation of e.s.d.'s in distances, angles and torsion angles; correlations between e.s.d.'s in cell parameters are only used when they are defined by crystal symmetry. An approximate (isotropic) treatment of cell e.s.d.'s is used for estimating e.s.d.'s involving l.s. planes.
Refinement. Refinement of *F*^2^ against ALL reflections. The weighted *R*-factor *wR* and goodness of fit *S* are based on *F*^2^, conventional *R*-factors *R* are based on *F*, with *F* set to zero for negative *F*^2^. The threshold expression of *F*^2^ > σ(*F*^2^) is used only for calculating *R*-factors(gt) *etc*. and is not relevant to the choice of reflections for refinement. *R*-factors based on *F*^2^ are statistically about twice as large as those based on *F*, and *R*- factors based on ALL data will be even larger.

### Fractional atomic coordinates and isotropic or equivalent isotropic displacement parameters (Å^2^)

**Table:**

	*x*	*y*	*z*	*U*_iso_*/*U*_eq_	
La1	0.0000	0.234447 (14)	0.341713 (14)	0.01947 (3)	
Cl1	0.10789 (2)	−0.09170 (6)	0.32281 (3)	0.03634 (12)	
C1	0.18755 (10)	0.3315 (3)	0.40149 (12)	0.0293 (4)	
C2	0.17044 (10)	0.4278 (3)	0.26382 (12)	0.0290 (3)	
N1	0.24102 (12)	0.2964 (3)	0.45518 (14)	0.0459 (5)	
H1	0.2283	0.2650	0.5022	0.055*	
H2	0.2882	0.3052	0.4427	0.055*	
N2	0.21433 (7)	0.3826 (2)	0.32868 (11)	0.0344 (4)	
H3	0.2628	0.3870	0.3229	0.041*	
N3	0.20884 (11)	0.4877 (3)	0.20138 (13)	0.0445 (4)	
H4	0.1851	0.5181	0.1591	0.053*	
H5	0.2575	0.4963	0.2032	0.053*	
O1	0.11900 (8)	0.3202 (3)	0.41736 (8)	0.0333 (3)	
O2	0.10047 (7)	0.41314 (19)	0.26393 (8)	0.0298 (3)	
O3	0.0000	0.0881 (4)	0.47616 (14)	0.0488 (7)	
H6	0.0406	0.0729	0.5026	0.059*	
O4	0.0000	0.1452 (4)	0.19698 (13)	0.0384 (5)	
H7	0.0377	0.0935	0.1750	0.046*	
O5	0.0000	0.5850 (3)	0.39200 (13)	0.0388 (5)	
H8	−0.0323	0.6527	0.3843	0.047*	
Cl2	0.36579 (3)	0.51610 (15)	0.08766 (5)	0.04627 (14)	
O6	0.5000	0.1924 (5)	0.17243 (18)	0.0617 (7)	
H9	0.4686	0.1472	0.2081	0.074*	
H10	0.5000	0.1363	0.1273	0.074*	
O7	0.39583 (14)	−0.0187 (4)	0.06811 (12)	0.0695 (6)	
H11	0.3960	−0.1404	0.0560	0.083*	
H12	0.3976	0.0476	0.0265	0.083*	

### Atomic displacement parameters (Å^2^)

**Table:**

	*U*^11^	*U*^22^	*U*^33^	*U*^12^	*U*^13^	*U*^23^
La1	0.01375 (4)	0.02565 (5)	0.01902 (5)	0.000	0.000	0.00102 (8)
Cl1	0.01972 (15)	0.03484 (19)	0.0545 (3)	0.00363 (13)	0.00053 (17)	−0.00126 (18)
C1	0.0219 (8)	0.0309 (9)	0.0351 (9)	−0.0001 (6)	−0.0063 (7)	−0.0041 (7)
C2	0.0240 (7)	0.0303 (8)	0.0325 (9)	−0.0042 (6)	0.0059 (7)	−0.0006 (7)
N1	0.0305 (9)	0.0593 (11)	0.0479 (11)	0.0024 (8)	−0.0163 (9)	0.0009 (10)
N2	0.0164 (5)	0.0455 (7)	0.0412 (11)	−0.0021 (5)	0.0022 (6)	0.0039 (7)
N3	0.0322 (9)	0.0584 (11)	0.0430 (10)	−0.0067 (8)	0.0141 (8)	0.0071 (9)
O1	0.0228 (6)	0.0510 (8)	0.0260 (6)	−0.0028 (6)	−0.0025 (5)	−0.0012 (6)
O2	0.0211 (6)	0.0395 (7)	0.0288 (6)	−0.0032 (5)	0.0006 (5)	0.0062 (6)
O3	0.0248 (10)	0.086 (2)	0.0352 (13)	0.000	0.000	0.0282 (12)
O4	0.0265 (10)	0.0609 (15)	0.0279 (10)	0.000	0.000	−0.0113 (10)
O5	0.0301 (10)	0.0299 (9)	0.0564 (14)	0.000	0.000	−0.0004 (9)
Cl2	0.03018 (19)	0.0726 (4)	0.0360 (2)	0.0037 (3)	−0.0008 (3)	−0.0124 (2)
O6	0.076 (2)	0.0640 (16)	0.0453 (16)	0.000	0.000	0.0068 (14)
O7	0.0972 (16)	0.0730 (12)	0.0384 (10)	0.0050 (13)	−0.0051 (10)	0.0115 (10)

### Geometric parameters (Å, °)

**Table:**

La1—O3	2.503 (2)	C2—N2	1.385 (3)
La1—O2	2.5313 (13)	N1—H1	0.8600
La1—O2^i^	2.5313 (12)	N1—H2	0.8600
La1—O1	2.5318 (14)	N2—H3	0.8600
La1—O1^i^	2.5318 (14)	N3—H4	0.8600
La1—O4	2.542 (2)	N3—H5	0.8600
La1—O5	2.562 (2)	O3—H6	0.8522
La1—Cl1^i^	2.9606 (4)	O4—H7	0.8418
La1—Cl1	2.9606 (4)	O5—H8	0.7477
C1—O1	1.241 (2)	O6—H9	0.8786
C1—N1	1.336 (3)	O6—H10	0.8615
C1—N2	1.374 (3)	O7—H11	0.8631
C2—O2	1.237 (2)	O7—H12	0.8446
C2—N3	1.327 (3)		
			
O3—La1—O2	132.46 (4)	O2—La1—Cl1	82.10 (3)
O3—La1—O2^i^	132.46 (4)	O2^i^—La1—Cl1	139.57 (4)
O2—La1—O2^i^	88.78 (6)	O1—La1—Cl1	72.56 (4)
O3—La1—O1	68.14 (5)	O1^i^—La1—Cl1	139.86 (4)
O2—La1—O1	64.78 (4)	O4—La1—Cl1	73.20 (5)
O2^i^—La1—O1	137.12 (5)	O5—La1—Cl1	138.709 (15)
O3—La1—O1^i^	68.14 (5)	Cl1^i^—La1—Cl1	79.930 (17)
O2—La1—O1^i^	137.12 (5)	O1—C1—N1	121.8 (2)
O2^i^—La1—O1^i^	64.78 (4)	O1—C1—N2	123.21 (16)
O1—La1—O1^i^	111.87 (7)	N1—C1—N2	115.01 (18)
O3—La1—O4	142.27 (9)	O2—C2—N3	122.33 (19)
O2—La1—O4	67.01 (5)	O2—C2—N2	122.51 (17)
O2^i^—La1—O4	67.01 (5)	N3—C2—N2	115.16 (17)
O1—La1—O4	123.41 (3)	C1—N1—H1	120.0
O1^i^—La1—O4	123.41 (3)	C1—N1—H2	120.0
O3—La1—O5	94.19 (9)	H1—N1—H2	120.0
O2—La1—O5	73.56 (5)	C1—N2—C2	125.93 (14)
O2^i^—La1—O5	73.56 (5)	C1—N2—H3	117.0
O1—La1—O5	67.03 (4)	C2—N2—H3	117.0
O1^i^—La1—O5	67.03 (4)	C2—N3—H4	120.0
O4—La1—O5	123.54 (8)	C2—N3—H5	120.0
O3—La1—Cl1^i^	78.13 (5)	H4—N3—H5	120.0
O2—La1—Cl1^i^	139.57 (4)	C1—O1—La1	135.26 (12)
O2^i^—La1—Cl1^i^	82.10 (3)	C2—O2—La1	137.53 (12)
O1—La1—Cl1^i^	139.86 (4)	La1—O3—H6	122.3
O1^i^—La1—Cl1^i^	72.56 (4)	La1—O4—H7	122.3
O4—La1—Cl1^i^	73.20 (5)	La1—O5—H8	121.9
O5—La1—Cl1^i^	138.709 (15)	H9—O6—H10	117.3
O3—La1—Cl1	78.13 (5)	H11—O7—H12	108.9
			
O1—C1—N2—C2	−0.1 (3)	Cl1^i^—La1—O1—C1	103.6 (2)
N1—C1—N2—C2	−179.32 (19)	Cl1—La1—O1—C1	54.6 (2)
O2—C2—N2—C1	−5.1 (3)	N3—C2—O2—La1	159.25 (15)
N3—C2—N2—C1	174.93 (19)	N2—C2—O2—La1	−20.7 (3)
N1—C1—O1—La1	−149.81 (17)	O3—La1—O2—C2	21.4 (2)
N2—C1—O1—La1	31.0 (3)	O2^i^—La1—O2—C2	175.02 (16)
O3—La1—O1—C1	138.5 (2)	O1—La1—O2—C2	29.91 (18)
O2—La1—O1—C1	−34.7 (2)	O1^i^—La1—O2—C2	125.52 (18)
O2^i^—La1—O1—C1	−91.9 (2)	O4—La1—O2—C2	−119.5 (2)
O1^i^—La1—O1—C1	−167.88 (17)	O5—La1—O2—C2	101.81 (19)
O4—La1—O1—C1	−0.6 (2)	Cl1^i^—La1—O2—C2	−108.69 (18)
O5—La1—O1—C1	−116.7 (2)	Cl1—La1—O2—C2	−44.48 (18)

Symmetry codes: (i) −*x*, *y*, *z*.

### Hydrogen-bond geometry (Å, °)

**Table:**

*D*—H···*A*	*D*—H	H···*A*	*D*···*A*	*D*—H···*A*
N1—H1···Cl2^ii^	0.86	2.94	3.643 (3)	141
N1—H2···Cl1^iii^	0.86	2.83	3.574 (3)	145
N2—H3···Cl1^iii^	0.86	2.28	3.1399 (14)	173
N3—H4···O7^iii^	0.86	2.12	2.927 (3)	156
N3—H5···Cl2	0.86	2.75	3.3835 (18)	132
O3—H6···Cl2^ii^	0.85	2.28	3.1181 (16)	168
O4—H7···Cl2^iv^	0.84	2.32	3.1396 (17)	164
O5—H8···Cl1^v^	0.75	2.44	3.1566 (17)	160
O6—H9···O2^iv^	0.88	2.23	3.044 (3)	153
O6—H10···O7	0.86	2.35	2.941 (3)	126
O6—H10···O7^vi^	0.86	2.35	2.941 (3)	126
O7—H11···Cl2^vii^	0.86	2.48	3.264 (3)	151
O7—H12···O1^viii^	0.84	2.09	2.922 (2)	168

Symmetry codes: (ii) −*x*+1/2, −*y*+1/2, *z*+1/2; (iii) −*x*+1/2, *y*+1/2, *z*; (iv) −*x*+1/2, *y*−1/2, *z*; (v) −*x*, *y*+1, *z*; (vi) −*x*+1, *y*, *z*; (vii) *x*, *y*−1, *z*; (viii) −*x*+1/2, −*y*+1/2, *z*−1/2.

## References

[bb1] Brese, N. E. & O’Keeffe, M. (1991). *Acta Cryst.* B**47**, 192–197.

[bb2] Bruker (1999). *SMART*, *SAINT* and *SADABS* Bruker AXS Inc., Madison, Wisconsin, USA.

[bb3] Carugo, O., Poli, G. & Manzoni, L. (1992). *Acta Cryst.* C**48**, 2013–2016.

[bb4] Farrugia, L. J. (1997). *J. Appl. Cryst.* **30**, 565.

[bb5] Flack, H. D. (1983). *Acta Cryst.* A**39**, 876–881.

[bb6] Haddad, S. F. (1987). *Acta Cryst.* C**43**, 1882–1885.

[bb7] Haddad, S. F. (1988). *Acta Cryst.* C**44**, 815–818.

[bb8] Harrison, W. T. A. (2008*a*). *Acta Cryst.* E**64**, m619. 10.1107/S1600536808008659 PMC2961277 21202174

[bb9] Harrison, W. T. A. (2008*b*). *Acta Cryst.* E**64**, m620. 10.1107/S1600536808008660 PMC2961212 21202175

[bb10] Rogers, R. D., Rollins, A. N., Etzenhouser, R. D., Voss, E. J. & Bauer, C. B. (1993). *Inorg. Chem.* **32**, 3451–3462.

[bb11] Sheldrick, G. M. (2008). *Acta Cryst.* A**64**, 112–122. 10.1107/S0108767307043930 18156677

[bb12] Su, Y., Yang, L., Wang, Z., Jin, X., Weng, S., Yan, C., Yu, Z. & Wu, J. (2006). *Carbohydr. Res.* **341**, 75–83. 10.1016/j.carres.2005.07.025 16297898

